# Biological assessment of ozone therapy on experimental oral candidiasis in immunosuppressed rats

**DOI:** 10.1016/j.bbrep.2018.06.007

**Published:** 2018-07-11

**Authors:** Laila E. Amin

**Affiliations:** Oral Biology, Faculty of Dentistry, Mansoura University, 35516 El-Gomhoria Street, Egypt

**Keywords:** Candida, Ozone, Tongue, Immunosuppression, CD3

## Abstract

Immunosuppressive drugs can compromise local or general defense mechanisms and can lead to oral candidiasis. Ozone therapy has a wide range of applications in almost every field of dentistry. Its unique properties include immunostimulant, analgesic, antihypnotic, detoxicating, and antimicrobial actions. Sixty male healthy rats were immunosuppressed with dexamethasone in their drinking water one week before candida infection. The animals were divided into four equal groups. Rats of group 1 were kept without any manipulation and those of group II were given oral inoculums of C. albicans on the dorsal surface of the tongue. Group III rats were handled as group II and instead the rats were treated by daily mycostatin drops local applicator as a routine treatment. Meanwhile, group IV rats were handled as group II and instead the rats were received daily intraperitoneal injection of 1 cm^3^ of ozone oxygen gas mixture with concentration of ozone 70 μg/cm^3^. After two weeks, all rats were euthanized and tongue specimens were prepared for histological staining with Haematoxylin & Eosin and CD3 immunohistochemical staining. Histological examination revealed that treatment with ozone therapy lead to gradual decrease in lingual papillary atrophy and invasion of candida yeast. Immunohistochemical study showed significant decrease in CD3 counting. We can conclude that ozone acts as an excellent fungicidal agent also, ozone is capable of alerting the immune system.

## Highlights

• Immunosuppressive drugs can compromise local or general defense mechanisms and lead to oral candidiasis.
• Ozone therapy has a wide range of applications in many fields of dentistry.
• Ozone application has many beneficial effects on the oral tissues comprising remission of various mucosal alterations.

## 1 Introduction

The epidemiological surveys indicate that Candida organisms are considered as commensals in the oral cavities of approximately 40% of healthy subjects [1] and that Candida albicans specifically is carried as a commensal organism in the mouths of approximately one-third of the population [2]. The opportunistic fungus C. albicans is a major cause of oral and esophageal infections in immunocompromised patients [3], [4] and affects up to 90% of patients with human immunodeficiency virus infection or AIDS [5]. This opportunistic human pathogen preferentially causes invasive and disseminated infections in patients with defective phagocytic defenses and serious mucocutaneous infection in patients with deficiencies in T-cell function. Phagocytes appear to protect the host from fungal colonization even in the absence of adaptive immune mechanisms, while as-yet-undefined T-cell-dependent factors seem necessary for the control of C. albicans on body surfaces [6].

As a consequence of this, the expression of C. albicans virulence in the oral cavity is strongly correlated with impairment of the immune system [2], [7]. Other conditions predisposing individuals to oral C. albicans infection include hyposalivation [6], [8], diabetes mellitus, prolonged use of antibiotics or immunosuppressive drugs, and poor oral hygiene [7], [9].

For a long time, polyene drugs including Amphotericin and Nystatin were the only therapeutic options for invasive fungal infections. The still unacceptably high morbidity rate associated with some resistant mycosis indicates that alternatives to existing therapeutic options are needed [10]. Ozone (O3) is a natural gaseous molecule made up of three oxygen atoms [11]. Ozone is heavier than air and hence it falls downward to earth from such high altitudes [12]. It cleanses the air and combines with any pollutant that it comes in contact. This is earth’s natural way of self-cleansing [13]. Ozone therapy can be defined as a versatile bio-oxidative therapy in which oxygen/ozone is administered via gas or dissolved in water or oil base to obtain therapeutic benefits [14]. Ozone therapy has a wide range of applications in treating various diseases owing to its unique properties including antimicrobial, immunostimulant, analgesic, antihypnotic, detoxicating, bioenergetic and biosynthetic actions [11].

Therefore, the aims of the present study to developed an experimental model in rats with impaired immune function and a stable microbial population in the oral cavity in order to evaluate the efficacy of ozone therapy against experimental oral C. albicans infection. The methods of evaluations were histological investigation and immunohistochemical expression for T-lymphocytes (CD3).

## 2 Materials and methods

### 2.1 Animals

60 adult male rats weighing 150–200 gm were used in this study. All experimental procedures were performed under approved protocol of ethical committee, Faculty of dentistry, University of Mansoura, Egypt. They were kept in individual cage and receive standard food for rodents and tap water. A light controlled room (12:12-h) light/ dark cycle and temperature (± 22 °C) and relative humidity (65–70%) will be kept constant.

### 2.2 Oral candidiasis and immunosuppression in rats

An animal model of oral candidiasis was induced basically as reported by Jones et al., [15] with some modifications. The rats were immunosuppressed, starting 1 week before experimental infection and continuing throughout the experiment, by administration of dexamethasone (Fortecortin; Merck Laboratories, Madrid, Spain) in the drinking water at a dose of 0.5 mg/l. Also, a 0.1% aqueous solution of tetracycline hydrochloride (Terramicine; Pfizer Laboratories, Alcobendas, Spain) was given to the rats, beginning 7 days before infection. The concentration of tetracycline hydrochloride were reduced to 0.01% on the day of infection and maintained throughout the experiment. A suspension of C.albicans, containing 5 × 108 viable cells/ml was prepared according to Reed et al. [16]. The suspension of C. albicans (0.2 ml) was dripped into the mouth of the rat with the aids of a 1 ml syringe and 30 × 8 mm blunt needle. Then the inoculum was spread on the tongue dorsum with a swab previously soaked in the suspension. The rats did not receive water for at least 1 h after inoculation. This procedure was repeated for 3 consecutive days. Establishment of C. albicans infection was evaluated by swabbing the inoculated oral cavity with sterile cotton applicator 6 days after inoculation, followed by plating on yeast extract- peptone- dextrose agar [4].

### 2.3 Ozone therapy

Ozone was generated by an ozone generator system (EXT120- T). It is a medical oxygen-fed ozone generator for ultra pure medical applications (Longevity resources Inc., Canada). Ozone obtained from medical grade oxygen was used immediately and it represented only about 3% of the gas (O2-O3) mixture. It produces 1–120 l g/ml ozone concentrations. The O3 concentration was measured by using an UV spectrophotometer at 254 nm as recommended by the Standardization Committee of the International Ozone Association. The ozone dose is the product of the O3 concentration (expressed as mg/l) by the gas (O2-O3) volume [17].

### 2.4 Experimental design

After induction of immunosuppression, the animals were randomized and assigned to groups of four.

Group I: rats were kept without any interventions. Group II: rats were given oral inoculums of C. albicans on the dorsal surface of the tongue. Group III: rats were subjected to C.albicans and treated by daily with mycostatin drops local applicator as a routine treatment. Group IV: Rats were given oral inoculums of C.albicans on the tongue dorsal surface and rats received daily intraperitoneal injection of 1 cm^3^ of ozone oxygen gas mixture with concentration of ozone 70 μg/cm^3^ [18].

Rats of different groups were sacrificed by cervical dislocation after two week. Tongue specimen was prepared for histological staining done with Haematoxylin & Eosin and immunohistochemical expression for CD3.

Statistical analysis: Data was analyzed using Statistical Package for Social Science software computer program version 23(SPSS, Inc., Chicago, IL, USA). Data was presented in mean and standard deviation. One way analysis of variance (ANOVA) and post-hoc tukey statistical tests were used for comparing quantitative means of the four groups. P value less than 0.05 was considered statistically significant.

## 3 Results

### 3.1 Gross observations

All infected and untreated animals had clinically manifest lesions of the lingual mucosa, consisting of patchy areas of smooth mucosa and well-delimited atrophic areas on the dorsum of the tongue. These lesions were mainly distributed surrounding the conical papillae of the tongue.

### 3.2 Haematoxylin and Eosin stain result

The histological examination revealed the following

**Fig. 1:**
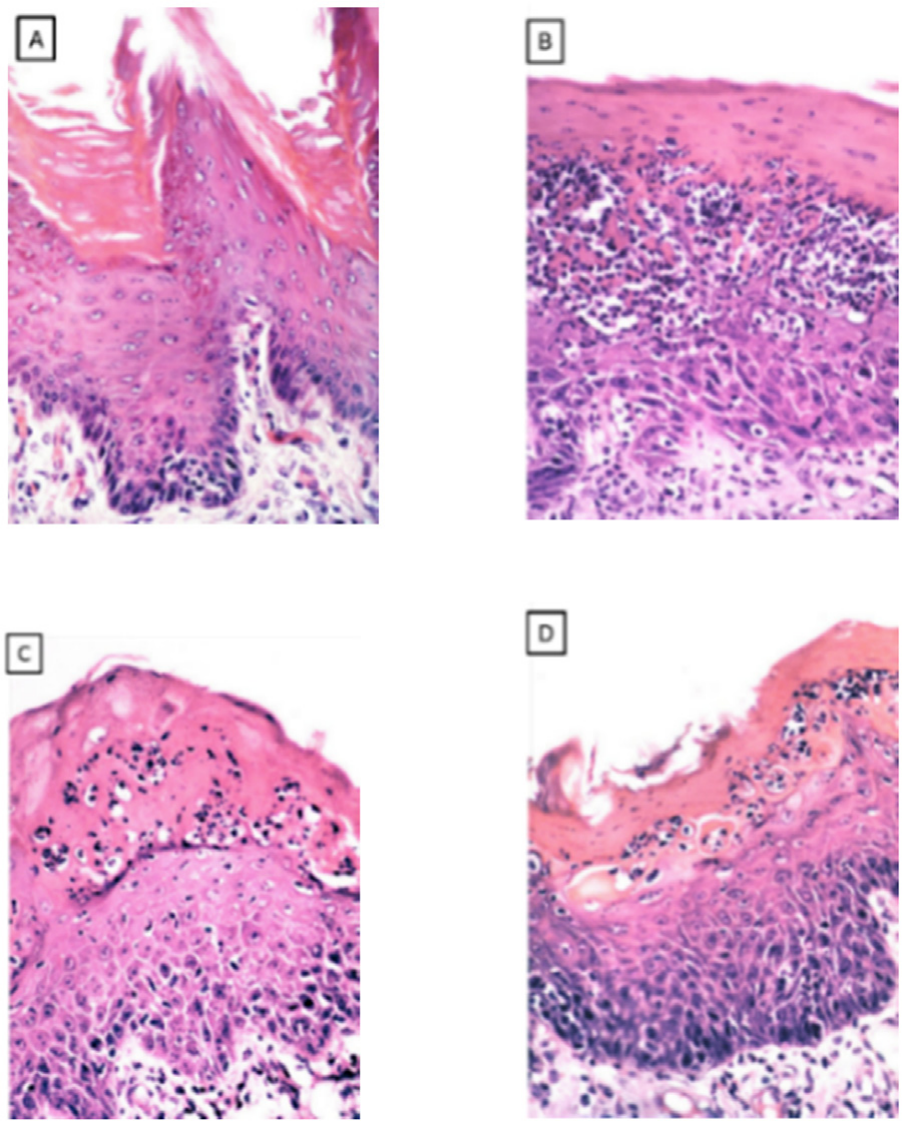
(A) group I showed the lingual mucosa with stratified squamous epithelium. (B) group II showed lingual papillary atrophy with extensive invasion of tangled masses of candida yeasts infiltration, (C) group III showed infiltration of the superficial layers with candida yeasts of the atrophied lingual epithelium, (D) thickened spinous cell layer with acanthosis crowded disorganized basal cell layer and aggregation of inflammatory cells and dilated blood vessels (H&E stain × 400).

Group I: the tongue showed normal stratified squamous epithelium overlying lamina propria (Fig. 1A).

Group II: showed marked lingual papillary atrophy with extensive invasion of tangled masses of candida yeasts infiltration, forming intraepithelial microabcesses accompanied with hyperkeratosis. The lamina propria with its connective tissue papilla showed discrete aggregation of inflammatory cells with dilated blood vessels (Fig. 1B).

Group III: showed infiltration of the superficial layers with candida yeasts of the atrophied lingual epithelium, crowded disorganized basal cell layer Intensive inflammatory cells in the lamina propria with dilated blood vessels (Fig. 1C).

Group IV: showed reduced infiltration of the superficial layers with candida yeast, thickened spinous cell layer and aggregation of inflammatory cells and dilated blood vessels (Fig. 1D).

### 3.3 Immunohistochemical results

**Fig. 2:**
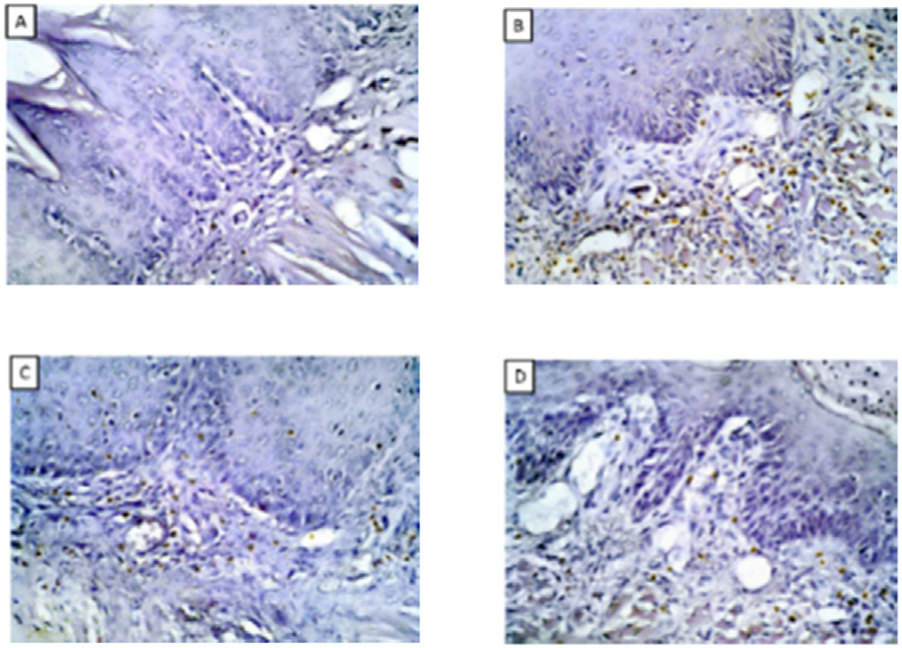
(A) CD3 immunohistochemical stain showed negative immunoreaction in group I, (B) intense immunoreaction in group II, (C, D) moderate in group III, IV (IHC × 400).

**Fig. 3:**
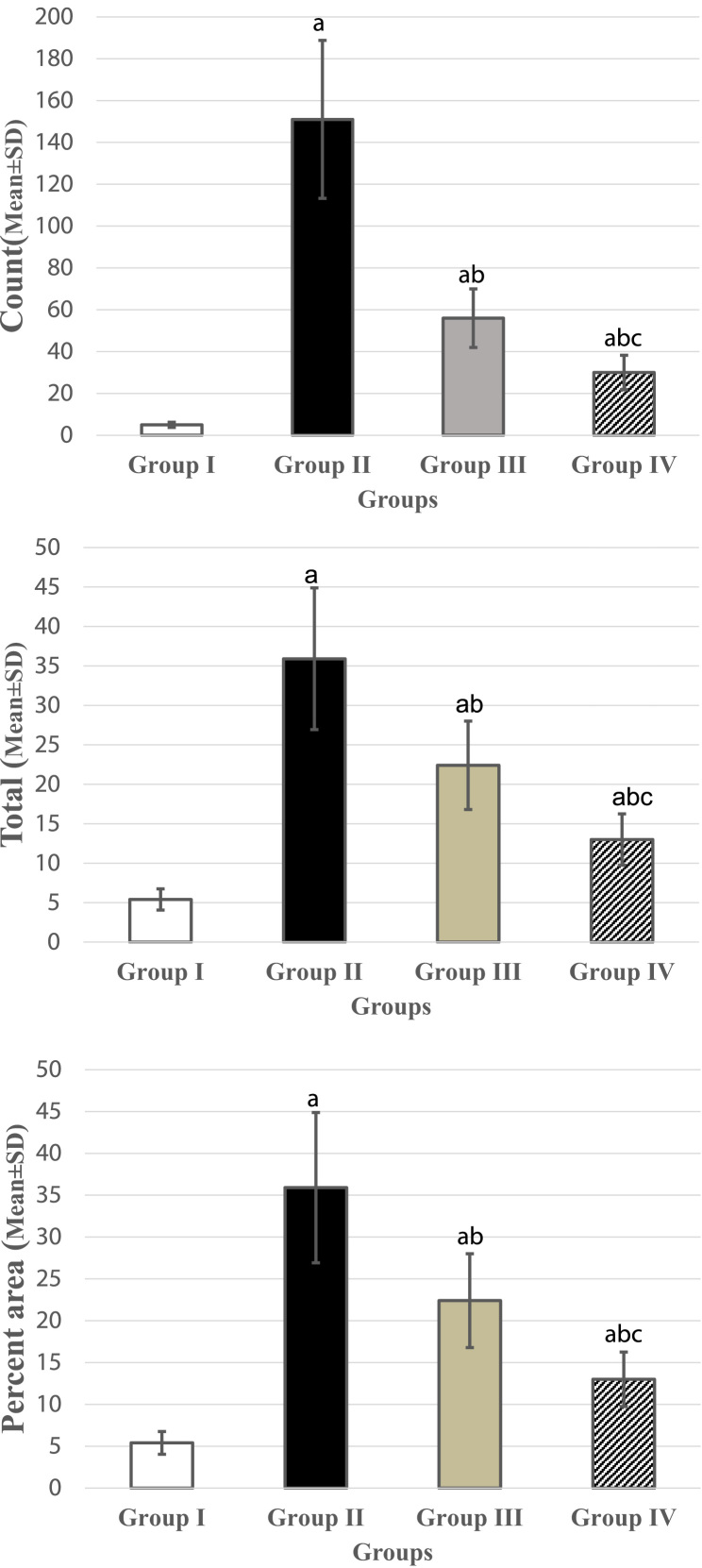
(A): Chart represent the difference in CD3 count (Mean ± SD). (B): Chart represents the difference in CD3 total number (Mean ± SD). (C): Chart represents the difference in CD3% area (Mean ± SD).

Group I: showed negative immunoreactivity for CD3, group II: showed positive intense positive immunoreaction in the lamina propria, moderate reaction in group III, group IV (Table 1, Fig. 2) (Fig. 3).

**Table 1:** Comparisons of count , total and percent area among different groups.

		**Group I**	**Group II**	**Group III**	**Group IV**	**P**
**Count**	Mean	5.0	151.0^a^	56.00^a^, ^b^	30.00^a^, ^b^, ^c^	< 0.001*
	±SD	1.25	37.75	14.00	8.25	
**Total**	Mean	93.00	420.0^a^	315.0^a^, ^b^	190.0^a^, ^b^, ^c^	< 0.001*
	±SD	23.25	105.0	78.75	62.50	
**Percent area**	Mean	5.40	35.90^a^	22.40^a^, ^b^	13.00^a^, ^b^, ^c^	< 0.001*
	±SD	1.35	8.98	5.60	3.25	

P: Probability.

Test used: One way ANOVA followed by post-hoc tukey.

* Significance < 0.05.

a Significance relative to Group I.

b Significance relative to Group II.

c Significance relative to Group III.

## 4 Discussion

The immunocompromised patients receiving immunosuppressive or anticancer therapy are most susceptible to an increased incidence of opportunistic mycoses. Although oral candidiasis is not a life-threatening disease, the sustained immunosuppression in these patients promote the recurrence of infection. Children and adolescents with a compromised or suppressed immune status are particularly susceptible to the development of oral candidiasis [3].

The present immunosuppressed-animal model appears to more closely as like as the condition seen in clinical settings. Furthermore, the administration of systemic antimicrobials, particularly tetracycline, is widely used to facilitate the development of candidiasis in the rat oral cavity [15], [19].

This study reported that ozone when administered therapeutically to immunosuppressed rats with oral candidiasis, it effectively reduce organism-mediated oral cavity injury. Histological examination showed reduced infiltration of the superficial layers with candida yeasts of the atrophied lingual epithelium. That comes in agreement with Tiwari et al., who reported that Ozone application has many beneficial effects on the oral tissues comprising remission of various mucosal alterations, improve wound healing and increased turnover rate of oral cells [11].

Ozone causes inactivation of bacteria, viruses, fungi, yeast and protozoa. It disrupts the integrity of the bacterial cell envelope by oxidation of phospholipids and lipoproteins. Ozone at low concentration of 0.1 ppm, is sufficient to inactivate bacterial cells including their spores [20]. In fungi, O3 inhibits cell growth at certain stages, budding cells being the most sensitive [21].

Huth et al., evaluated the biocompatibility of gaseous and aqueous forms of ozone in relation to the established antimicrobials. They reported that ozone is a potential antiseptic agent and the aqueous form showed less cytotoxicity than gaseous ozone or established antimicrobials (chlorhexidine digluconate 2%, 0.2%; sodium hypochlorite- 5.25%, 2.25%; hydrogen peroxide 3%) under most conditions. Therefore, aqueous ozone fulfills optimal cell biological characteristics in terms of biocompatibility for oral application [22].

The ability of candidal microorganisms to cause human infectious disease relates more probably to the immunological status of the host than to any obvious virulence factors [23]. Owing to kosonen et al., who reported significant increase in CD3 with candida infection [24]. The current immunohistochemical analysis for CD3 showed significant difference among the experimental groups. In the same context, Bocci .,et al stated that Ozone is a strong oxidizer of cell walls and cytoplasmatic membranes of bacteria and is considered to be one of the best bactericidal, antiviral, and antifungal agents [25]. Additionally, when Ozone administered at a concentration of between 30 and 55 g/cc causes the greatest increase in the production of interferon and the greatest output of tumor necrosis factor and interleukin-2 that launches an entire cascade of subsequent immunological reactions [26].

Tiwari et al., reported that ozone application has various beneficial effects on the oral tissues including remission of various mucosal alterations, enhanced wound healing and increased turnover rate of oral cells [11]. also, Huth et al., reported that ozone is a potential antiseptic agent and the aqueous form showed less cytotoxicity than gaseous ozone or established antimicrobials under most conditions. Therefore, aqueous ozone fulfills optimal cell biological characteristics in terms of biocompatibility for oral application [22].

The germicidal action of ozone is based on the oxidative transitory stress, which is deadly for the microorganism due to the weakness of its antioxidant defense system [27], [28]. Microorganisms lack enzymes such as catalase and glutathione peroxidase which conform the cellular defense system capable to challenge and neutralize the oxidative action of ozone. Ozone is capable of stimulating a certain number of cells of the immune system [29], [30]. As a result, these cells may release small amounts of immunostimulating and immunosuppressing cytokines [31], [32]

## 5 Conclusion

The current study suggested that ozone therapy has a possible benefit in attenuating the healing response of candida infection in immunosuppressed rats. Although further investigations and comparative toxicity profile studies are needed to confirm ozone therapy as very promising and effective antifungal agents in human candida infections.
